# Unravelling the Phases of Melamine Formaldehyde Resin Cure by Infrared Spectroscopy (FTIR) and Multivariate Curve Resolution (MCR)

**DOI:** 10.3390/polym12112569

**Published:** 2020-11-02

**Authors:** Stephanie Weiss, Regina Seidl, Waltraud Kessler, Rudolf W. Kessler, Edith M. Zikulnig-Rusch, Andreas Kandelbauer

**Affiliations:** 1 Kompetenzzentrum Holz GmbH, Altenberger Straße 69, 4040 Linz, Austria c/o Area 3, Klagenfurter Straße 87–89, 9300 St. Veit an der Glan, Austria; s.weiss@wood-kplus.at (S.W.); r.seidl@wood-kplus.at (R.S.); e.zikulnig-rusch@wood-kplus.at (E.M.Z.-R.); 2 Kessler ProData GmbH, Kaiserstraße 66, 72764 Reutlingen, Germany; rwkessler@t-online.de (W.K.); rudolf.kessler@reutlingen-university.de (R.W.K.); 3 Lehr und Forschungszentrum Process Analysis and Technology (LFZ PA&T), School of Applied Chemistry, Reutlingen University, Alteburgstraße 150, D-72762 Reutlingen, Germany

**Keywords:** melamine formaldehyde resin, curing, infrared spectroscopy, multivariate curve resolution

## Abstract

Here, we study resin cure and network formation of solid melamine formaldehyde pre-polymer over a large temperature range via dynamic temperature curing profiles. Real-time infrared spectroscopy is used to analyze the chemical changes during network formation and network hardening. By applying chemometrics (multivariate curve resolution, MCR), the essential chemical functionalities that constitute the network at a given stage of curing are mathematically extracted and tracked over time. The three spectral components identified by MCR were methylol-rich, ether linkages-rich and methylene linkages-rich resin entities. Based on dynamic changes of their characteristic spectral patterns in dependence of temperature, curing is divided into five phases: (I) stationary phase with free methylols as main chemical feature, (II) formation of flexible network cross-linked by ether linkages, (III) formation of rigid, ether-cross-linked network, (IV) further hardening via transformation of methylols and ethers into methylene-cross-linkages, and (V) network consolidation via transformation of ether into methylene bridges. The presented spectroscopic/chemometric approach can be used as methodological basis for the functionality design of MF-based surface films at the stage of laminate pressing, i.e., for tailoring the technological property profile of cured MF films using a causal understanding of the underlying chemistry based on molecular markers and spectroscopic fingerprints.

## 1. Introduction

Melamine resin surfaces play an exceptionally important role in the surface treatment of wood-based materials [1]. Almost three-quarters of all engineered wood-based panels such as particle board, medium density fiberboard (MDF) or hardboard are coated with melamine resin impregnated papers [2]. Such decorative laminates are used in a wide variety of products. These range from furniture [3,4], highly abrasion-resistant flooring [5], chemical-resistant laboratory tables, and steam-resistant working surfaces to antibacterial surfaces [6], durable exterior cladding and special applications such as anti-fingerprint surfaces [7], custom design surfaces [8,9], white boards for office use [7], drilling entry boards for the electronics industry or even self-healing surfaces [10]. Each product group has a specific property profile, especially with regard to the surface, which is tailored to fit the respective application.

The versatile range of properties can, in principle, mostly be covered by a single type of MF impregnating resin, whose surface properties can be adjusted accordingly by selecting the appropriate processing parameters [11]. The curing conditions in particular play a major role in this process. The degree of curing and the network density are, besides the used F/M ratio, mainly dominated by the pressing process [12,13]. The detailed chemistry of the specific thermoset network formed during curing is of particular importance with regard to the properties achieved [14,15]. Depending on how the curing of the resin is conducted in the process, the resulting surface properties can vary within wide limits [16]. Experience has shown that “well” cured MF films often show better performance in terms of hardness, gloss or chemical resistance. Poorly selected curing conditions such as, for instance, too high temperatures and/or too long pressing times may lead to inferior surface performance due to so-called “over-curing” (i.e., excessive condensation of the resin) with the result of excessive shrinkage, which can cause warping of the composite and cracks in the surface. In a previous study, we have shown that at different curing temperatures the curing process follows very different reaction trajectories [17]. These curing trajectories lead to different final states in the solid resin, that differ, for instance, in their hydrolytic stability. It is therefore important to set the ideal curing conditions for each product property profile [18].

Despite the important influence of the curing process on the final surface properties, there is unfortunately only limited scientific literature on this subject. In particular, the chemical processes that take place in the solid phase at high degrees of condensation during curing (for example in a press tool) are still practically unexplored. Therefore, it is currently not yet readily possible to build up the necessary technical know-how based on a scientific understanding required to tailor surface property profiles in a targeted manner, and much is mainly based on experience.

In the present work, we intend to contribute to closing this gap. Thermal curing of a spray-dried, solid melamine-formaldehyde resin pre-polymer was performed using a dynamic heating profile from room temperature to 200 °C. Curing was monitored using real-time in-situ FTIR spectroscopy. The spectral information was further processed by applying the chemometric tool “multivariate curve resolution, MCR”. This was done to extract spectral components from the evolving reaction spectra in order to reduce the data complexity and better understand the essential chemical changes taking place during resin cure.

## 2. Materials and Methods

### 2.1. Chemicals

Spray-dried melamine formaldehyde resin (Kauramin^®^ 773) was obtained as a white, fine powder from BASF (Ludwigshafen, Germany). It had a melamine-to-formaldehyde ratio of F/M = 1.6.

### 2.2. Resin Curing Experiments and Real-Time In Situ IR Spectroscopy

Curing experiments were performed directly on the attenuated total reflectance (ATR) cell of an FTIR spectrometer Bruker Tensor 27 (Bruker Optik GmbH, Ettlingen, Germany) which was equipped with an adjustable heating stage (“Golden Gate Heated Diamond Top Plate”) using defined heating ramps. During curing, infrared spectra were simultaneously recorded in real-time using the OPUS 2.7 software package. Samples of the spray dried MF powder resin were cured during heating from 30 to 200 °C with a heating rate of 2 °C min^−1^. This slow temperature increase rate was chosen to allow enough time for recording a sufficiently high number of infrared spectra to be able to follow the changes in the spectra with time at a high resolution. Spectra were recorded every minute in the range from 4000 to 600 cm^−1^ versus air as the background spectrum with a resolution of 4 cm^−1^ and a scan rate of 32 scans per spectrum. All curing experiments were performed three times and the average value of the resulting reaction spectra was used for further analysis.

### 2.3. Data Analysis—Multivariate Curve Resolution (MCR)

Multivariate curve resolution (MCR) of the IR spectra was performed using the Unscrambler^®^ X 10.4 software package (CAMO Software AS, Oslo, Norway). The spectral region from 1670 to 700 cm^−1^ was analyzed. The normalized (unit vector normalization) spectra were used for further chemometric analysis. During this study, for MCR procedures, non-negativity and closure were applied as constraints. No prior assumptions (e.g., pure component spectra) were made.

### 2.4. Differential Scanning Calorimetry

Thermograms were recorded using a differential scanning calorimeter DSC 822e by Mettler Toledo (Gießen, Germany) About 8 mg of the powdered resin sample were weighed into a high-pressure gold coated stainless steel crucible (30 µL) which was sealed and further subjected to a temperature gradient from 25 to 200 °C with a heating rate of 2 °C min^−1^.

### 2.5. Thermogravimetric Analysis

TGA measurements were carried out using a TA Instruments TGA Q5000 (TA Instruments, New Castle, DE, USA) device. Samples of the powdered resin of about 5 mg were weighed in platinum vessels. Nitrogen gas was used as flow medium with a flow rate of 25 mL min^−1^. The applied temperature profile ranged from 30 to 250 °C with a linear temperature increase rate of 2 °C min^−1^. All samples were measured twice. Weight loss curves were recorded and analyzed (differentials) using TA Instruments Universal Analysis 2000 package.

### 2.6. Dielectric Analysis

The measurements were performed in multi-frequency mode at 1000 Hz and 10 kHz on a DEA cure monitor Epsilon 230/1 (Netzsch Gerätebau GmbH, Selb, Germany). A printed paper sensor with a finger and a gap width of electrodes of 300 µm was used. The sensor was prepared for analysis by immersing it for about 10 s in an aqueous dispersion of the powdered resin containing 50 wt% solid resin and pre-drying it over night at room temperature. The impregnated sensor was then subjected to a temperature program from 30 to 180 °C with a heating rate of 3 °C min^−1^. The measurements were performed three times.

### 2.7. Rheology

Rheometer measurements of the powdered MF resin were performed with a Physica MCR 101 rheometer by Anton Paar (Graz, Austria) using the plate-plate system D-PP25 with a diameter of 25 mm. The temperature was increased from 30 to 180 °C with a linear heating rate of 2 °C min^−1^ using a PTD 200 temperature control unit. The measurement conditions were based on a series of preliminary experiments not detailed here. The deformation, γ, was set to 0.05% and a frequency, f, of 1 Hz was used. Due to the nature and the expansion of the sample, the normal force, F_N_, had to be altered from 10 to zero N with increasing temperature (10 N from 20 to 80 °C, linear decrease to 0 N from 80 to 120 °C, 0 N from 120 to 180 °C). A constant force of 10 N was set in the range up to 80 °C to ensure that there was always sufficient contact between sample and plate. However, to keep this force constant, the initial gap distance of d of 0.9 mm had to be adjusted during the measurement. The storage, G′, and loss, G″, moduli and the complex viscosity,∣η*∣, were recorded.

## 3. Results and Discussion

### 3.1. Structural Characterization of MF Resin during Cure via Real-Time Infrared Spectroscopy

**Figure 1:**
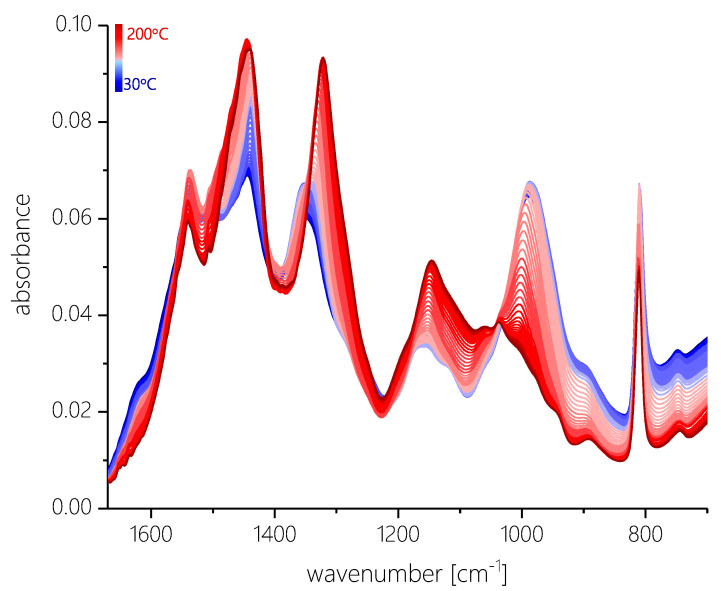
Infra-red spectra recorded during curing of the spray-dried MF-resin system Kauramin^®^773 at a heating rate of 2 °C min^−1^ using real-time in-situ FTIR spectroscopy.

While curing, in-situ FT infrared spectra were continuously recorded using ATR sampling technique. Figure 1 shows the infrared spectra recorded while the resin was cured.

A detailed band assignment of the used MF resin system was done in a previous study [19] which showed that the fingerprint area (about 1700 to 700 cm^−1^) provides a lot of information on the curing reaction. Hence, the focus of this study lies on analysis of the fingerprint region of the mid-IR spectra. The most important bands are briefly described in the following.

Melamine formaldehyde resins possess two absorbance bands due to vibrations of the triazine ring at about 1540 cm^−1^ and 810 cm^−1^. These absorbance bands stay nearly constant during the whole curing process whereas other bands undergo significant changes. The band shift of the cross-sensitive (or X-sensitive [20]) C_Ar_–N absorption band from about 1355 to 1320 cm^−1^ is due to the change of the substituents at the amine moiety resulting from the formation of cross-links during condensation. Consequently, a decrease in concentration of methylol functions (visible in IR as C–O stretching vibration at about 990 cm^−1^) is recorded as curing progresses. The spectral region from about 1200 cm^−1^ to 1000 cm^−1^ experiences changes as well. Within this region, C–O–C vibrations of ether moieties as well as other C_Ar_–N vibrations occur. For example, the rise of the asymmetric C–O–C vibration band (weak, at about 1030 cm^−1^) during the reaction is clearly visible. The vibrations of primary amine functions, whose concentration decreases during hardening, are present at about 1620 cm^−1^. CH_2_ groups are visible in the region from about 1500 to 1400 cm^−1^ but they may show signals at several positions within the fingerprint region [19,20].

### 3.2. Extraction of Component Spectra via Chemometric Data Analysis Using Multivariate Curve Resolution (MCR) and Identification of Spectral Components

The normalized spectra of the fingerprint area obtained from the real-time IR measurements were further used for the Multivariate Curve Resolution (MCR) calculations.

In MCR, deconvolution of the reaction spectra is performed, i.e., the spectral information of the complex reaction system is broken down into a small number of so called “spectral components”. These are invariable mathematical entities that are extracted from the data matrix comprising all reaction spectra obtained during the curing experiments. [21,22] They represent spectral fingerprints that are recognized by the algorithm as constant patterns throughout all transformations at any stage of the transformations. [23,24] The characteristic changes of these spectral components with time during cure are analyzed.

In case of simple reaction mixtures, the spectral components can be identified with real chemical constituents of this mixture by comparing their spectral fingerprints with real absorbance patterns of actual chemicals. An unambiguous assignment of spectral single components to real, isolatable chemical compounds has already been demonstrated in the past with various systems [25,26,27]. These examples illustrate how powerful this method is in elucidating reaction mechanisms.

Naturally, in the case of a complicated mixture such as a thermosetting resin, the direct assignment of spectral components to real constituents is difficult or, even in principle, impossible [26]. Formaldehyde pre-polymers consist of complicated oligomer mixtures that easily contain dozens of different compounds of great chemical similarity. It is already known from liquid resin mixtures that the main chemical reactions taking place are based on a small number of different functional groups transforming into each other [28,29]. Due to their constant transformation and incorporation into larger oligomer fragments in a sequence of coupled equilibria, individual molecules quickly lose their identity and can no longer be tracked individually. At any rate, after reaching the gel point, it no longer makes sense at all to speak of distinctly different components, since the overall system is determined either by the presence of one single giant molecule that extends across the entire reaction volume (textbook definition of the “gel point”) or, maybe more realistically, a system of several or many polymer molecules interpenetrating each other. During completion of cure, this macromolecular network still undergoes further changes in the solid phase. If the chemistry at such very high degrees of conversion in the solid state is to be described, there are in principle no longer any sensibly definable chemical components into which the „mixture“ could be broken down.

Nevertheless, the present study shows that MCR can still be used to investigate such processes with advantage. It is not necessary to follow the fate of each individual molecule present in the pre-polymer over the reaction time in detail. In the curing of thermosetting polymers, the conversion of single molecules is of less interest than the evolution of the entire polymer system, i.e., the formation of the network as a whole and its essential chemical characteristics that determine its technological performance.

In the present study, the spectral components are no longer assigned to individual molecules, but instead to so-called “network components”, which are characterized by spectral fingerprints extracted by MCR. These (calculated) network components are already present at the beginning of the curing process and only change their relative proportions over the course of the curing reaction. In their entirety they produce the observed spectral pattern of the overall evolving thermosetting system. It is justified to speak of network components, since higher molecular weight oligomers are already present at the beginning of cure and form the so-called “pre-polymer” which contains already ether and methylene bridges in varying amounts between the melamine cores as a result of the manufacturing process that has been performed up to that point.

It is interesting that the most appropriate mathematical deconvolution of the dynamic reaction spectra yields only three of such independent network components despite the complexity of the studied system. Using three components, the total residuals (which represent the part of the data set that is not explained with the calculated model) are low (only about 10^−5^ to 10^−6^ absorbance units). The use of a larger number of spectral components does not lead to any significant improvement in accuracy. On the contrary, using a higher number of components, the total residual value increases again. This indicates that the chemistry of melamine resin cure is actually essentially determined by the mutual conversion of three spectroscopically distinguishable basic types of chemical functionalities.

**Figure 2:**
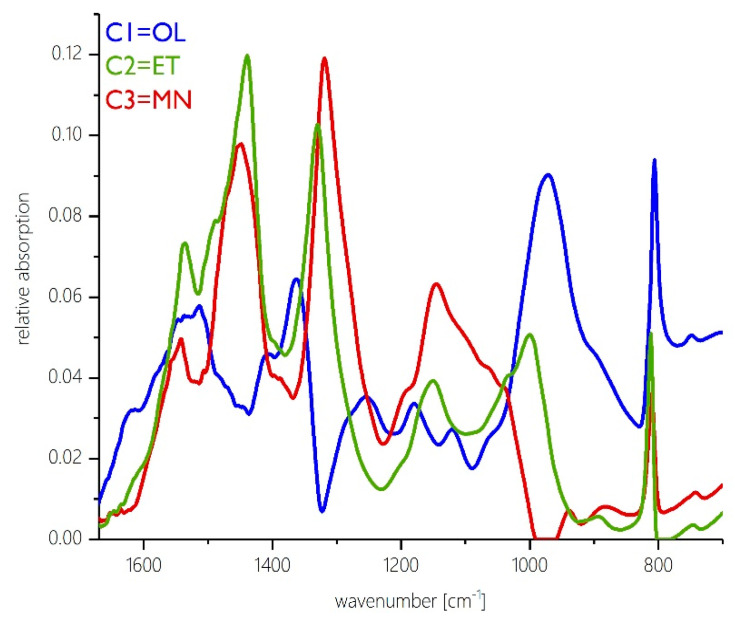
Spectral fingerprints extracted from the original reaction spectra via MCR representing the three invariable absorbance patterns OL (blue), ET (green) and MN (red) comprising the overall spectral information during MF resin cure.

The spectral fingerprints of the calculated network components are given in Figure 2.

For each spectral pattern, a detailed band assignment was performed.

The calculated spectrum of component 1 (which represents the methylol-rich network component “OL”) shows strong absorptions in the region of C–O vibrations of primary alcohols at about 980 cm^−1^ which can be attributed to methylol moieties. The X-sensitive C_Ar_–N band appears at about 1365 cm^−1^ which also indicates that methylol groups are predominantly attached to the amino functionalities. The area, that is attributed to primary amines at 1620 cm^−1^, shows absorptions as well. This component spectrum is very similar to the original IR spectra at the initial stage of resin cure in the beginning of the data acquisition.

Component 2 (which represents the ether-rich network component “ET”) shows still a band in the region of 1000 cm^−1^ but a shoulder of considerable intensity is present at about 1040 cm^−1^ which can be assigned to the C–O–C vibration of methylene-ether bonds. The C_Ar_–N band has changed its position to about 1345 cm^−1^. The C–H vibrations are in the area of 1500 to 1450 cm^−1^. Component 2 contains a considerable amount of methylene-ether bonds and it still contains a small amount of methylol moieties as well.

The spectrum of the third component (which represents the methylene-rich network component “MN”), however, lacks the C–O absorption of methylol groups completely. The C_Ar_–N band appears at lower wavenumbers compared to component 1 at about 1320 cm^−1^. This points out, that methylene bridges have formed. C–H vibrations at 1450 cm^−1^ are strongly present in this component spectrum. It strongly resembles the IR spectra measured towards the end of the reaction.

Comparison of the spectral patterns (Figure 2) shows that although the three components display distinctly different spectral features, practically all types of functional groups are present at all times in all three components over the entire period of curing time. A distinct spectroscopic separation into different chemical compounds is not possible and, as explained above, also not to be expected. However, the relative contributions of individual group vibrations differ from component to component. Therefore, according to the relative abundance of individual group vibrations, the three network components can be characterized according to the respective predominating functional groups. The three types of network components are classified as follows:

(1) Spectral component 1 shows an especially high density of signals that are characteristic for functional groups related to free methylol groups. Hence, in the following, it will be referred to as the methylol-rich or, in short, “OL” network component.
(2) Spectral component 2 displays an especially high proportion of group vibration frequencies that are typical of methylene ether functionalities. Hence, in the following it will be referred to as the ether-rich, or “ET” network component since it is presumably rich in ether linkages.
(3) Spectral component 3 contains a high proportion of functional groups related to methylene bridges. Therefore, in the following it will be referred to as the methylene-rich, or “MN” network component because it presumably contains a more than average proportion of methylene bridges.

### 3.3. Time Course of Spectral Components—Curing Profile

**Figure 3:**
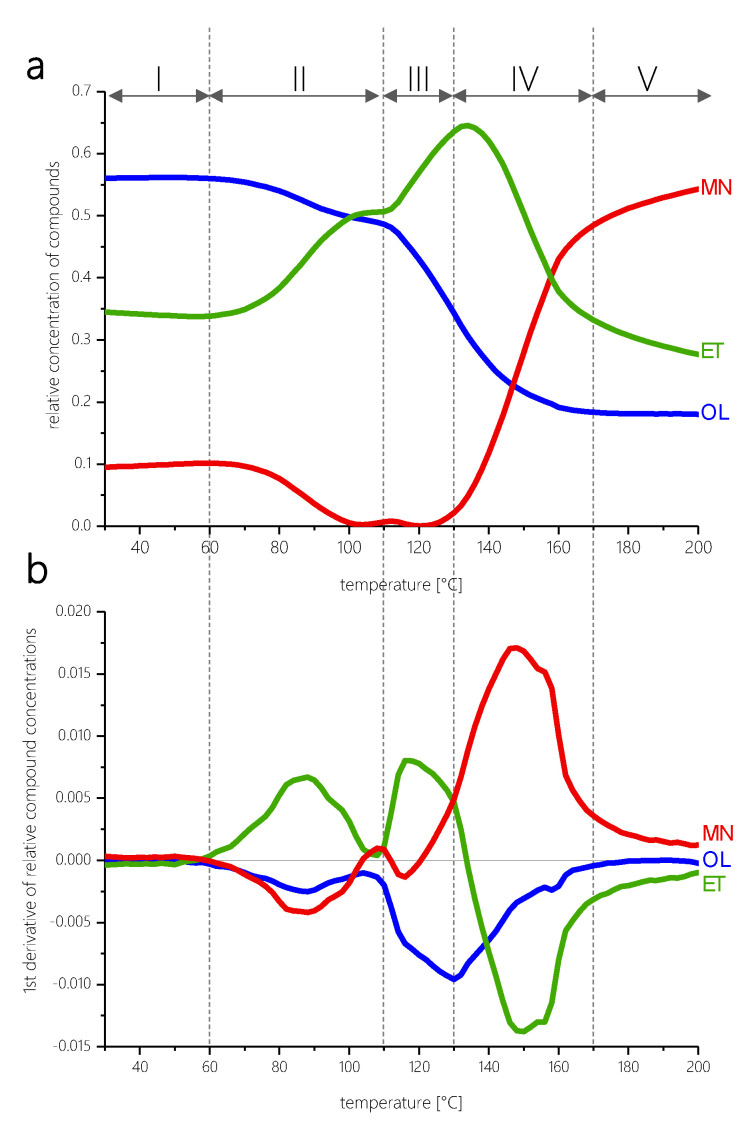
Spectroscopic concentration profiles. (**a**) Relative changes in proportions of the three identified spectral components OL, ET, and MN over temperature, and (**b**) the first derivative representing the relative rates of changes in dependence of the temperature. Five distinct phases of resin cure are indicated by Roman numerals.

Figure 3a shows the changes in relative proportions of the three network components (OL, ET, MN) during curing of the MF resin in dependence on the temperature. Figure 3b shows the first derivative of these spectroscopic concentration profiles as a function of temperature as well. From Figure 3a, several temperature ranges are discernable in which characteristic relative concentration ratios of the three network types are established as the temperature gradually increases. These temperature ranges are indicated in Figure 3 in Roman numerals.

The following five subsequent phases of curing can be identified as a function of temperature:

**PHASE I**—Temperature range from room temperature to around 60 °C

As long as subjected to a temperature <60 °C, the MF network present at this stage does not undergo any effective chemical conversions. The initial ratio between the three spectroscopic components remains unchanged and is defined by the initial state of the melamine-formaldehyde pre-polymer, which undergoes thermal curing. This initial state of the MF system is determined by the conditions applied during synthesis and any subsequent processing steps that may follow the synthesis, such as storage, impregnation or drying. Decisive factors determining the particular proportions between the three spectroscopic components in this initial stage are, among others, the molar ratio F/M with which the pre-polymer was synthesized, the reaction conditions used (pH, temperature, reaction time, etc.) [10] and whether, as in the present case, spray drying or other further treatments have been applied.

In the initial state, the OL component is the most important component in terms of relative quantity: the pre-polymer contains a very high content of free methylol functionalities. At the same time, it is comparatively poorly bridged in this phase. The ET component, which is mainly characterized by the vibration of functional groups attributable to ether bridges, is much less present and the MN component only plays a very minor role. No profound change in the chemical structure of the pre-polymer is observed in the low temperature regime <60 °C.

**PHASE II**—Temperature range from around 60 to 110 °C

Heating only leads to a change in the distribution between the characteristic functional groups once a minimum temperature of approx. 60 °C is exceeded. In this first phase of distinct chemical transformations in the temperature range between 60 and 110 °C, the relative proportion of methylene ether bridges increases sharply (increase in component concentration ET, Figure 3a). Thereby, a system containing more chemical bridges and cross-links is formed. The proportion of methylol groups significantly declines (decrease in OL, Figure 3a). The MN component initially present to a minor degree practically disappears completely. In simplified terms, the following two main transformations take place: (1) condensation of free methylol groups to ether bridges with the release of water (Equations (1) and (2)) hydrolysis of methylene bridges in the presence of water under formation of methylols (Equation (2)), which can partially continue to react according to (Equation (1)) via condensation:

(1) R-NH-CH_2_-OH + HO-CH_2_-NH-R ⇄ R-NH-CH_2_-O-CH_2_-NH-R + H_2_O

(2) H_2_O + R-NH-CH_2_-NH-R ⇄ R-NH-CH_2_-OH + H_2_N-R

**Figure 4:**
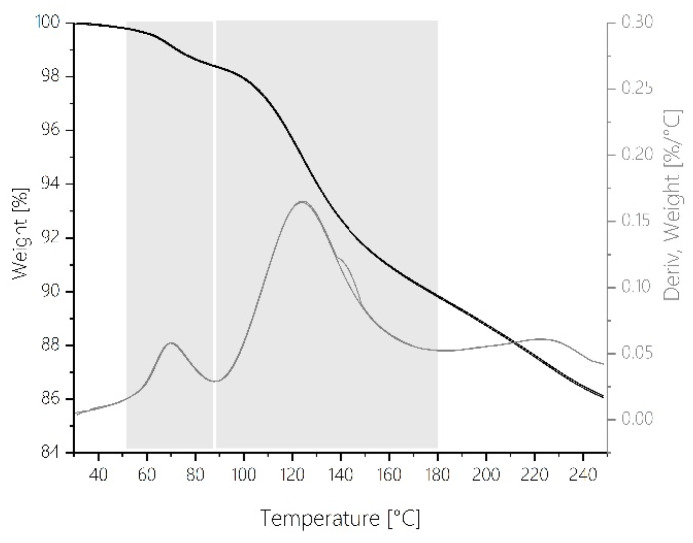
Weight changes during MF resin cure in dependence of temperature in absolute values and first derivative as recorded by thermogravimetric analysis using a temperature gradient of 2 °C min^−1^.

Thermogravimetric measurements on the same spray-dried MF pre-polymer using the same temperature program show that in this temperature range (from 60 to 90 °C) volatile low molecular weight substances are liberated such as water from the condensation reaction and residual moisture content as well as free formaldehyde based on the chemical equilibrium between bonded and free formaldehyde (Figure 4).

This vapor phase is mobile within the forming network. Under the reaction conditions of the ATR measurement (i.e., when high pressure is applied to press the sample onto the ATR crystal), it causes hydrolysis of bridged species. Although methylene bridges are, in principle, more stable than ether bridges, both types of bridges are hydrolyzed, but, according to the literature [30] ether bridges are more favorably formed again in this temperature range. Thus, in total, formation of ether bridges is favored over methylene bridge formation. By the water liberated during condensation of methylols to ether bridges, the equilibrium (Equation (2)) is shifted in the direction of the hydrolysis products and methylols are formed. The methylols released from hydrolysis are quickly removed from the equilibrium (Equation (2)) via condensation to ether bridges (Equation (1)), which also shifts Equation (2) in favor of the hydrolysis products.

The predominance of methylene ether-rich network components in this temperature regime is in good agreement with the literature. It was shown earlier that condensation at low temperature favors the formation of ether bridges over methylene bridges [30].

**Figure 5:**
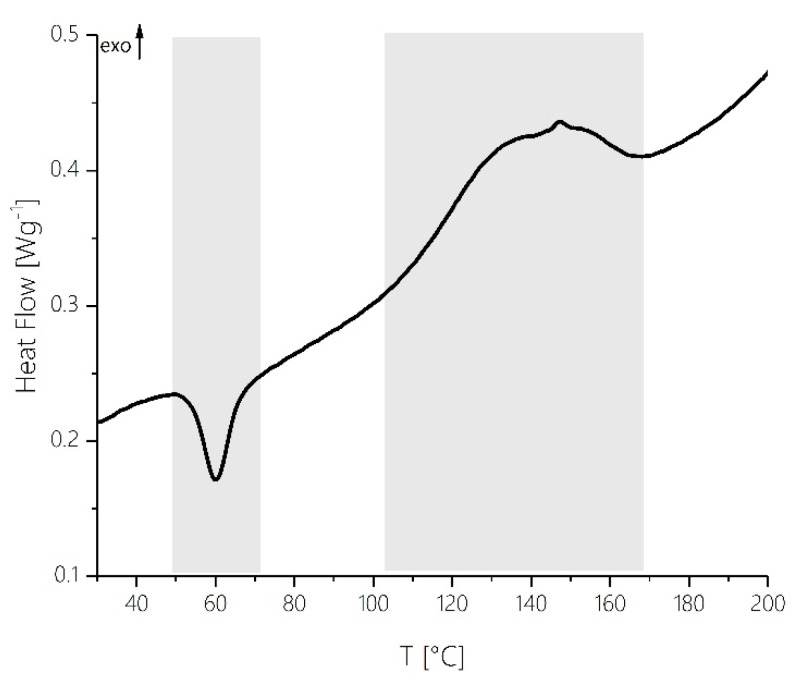
DSC trace recorded during MF resin cure in a temperature range from 30 to 200 °C at a heating rate of 2 °C min^−1^. Please note, that the reference signal was not subtracted from the sample signal.

Furthermore, morphological changes of the solid pre-polymer powder can be observed in this temperature range with the spray-dried MF system investigated here. The thermogram depicted in Figure 5 of a sample analyzed under high pressure conditions by dynamic DSC using the same temperature profile shows an endothermic heat transfer in the temperature interval between 50 and 70 °C. Since a high-pressure crucible was used for the measurement, this signal cannot be due to endothermal phase changes caused by evaporation of low molecular volatiles. These morphological changes only occur during the first heating cycle, and no signal is observed at this point when heating is repeated after intermediate cooling. With liquid MF resin samples, under analogous curing conditions no such heat transfer occurs at all.

**Figure 6:**
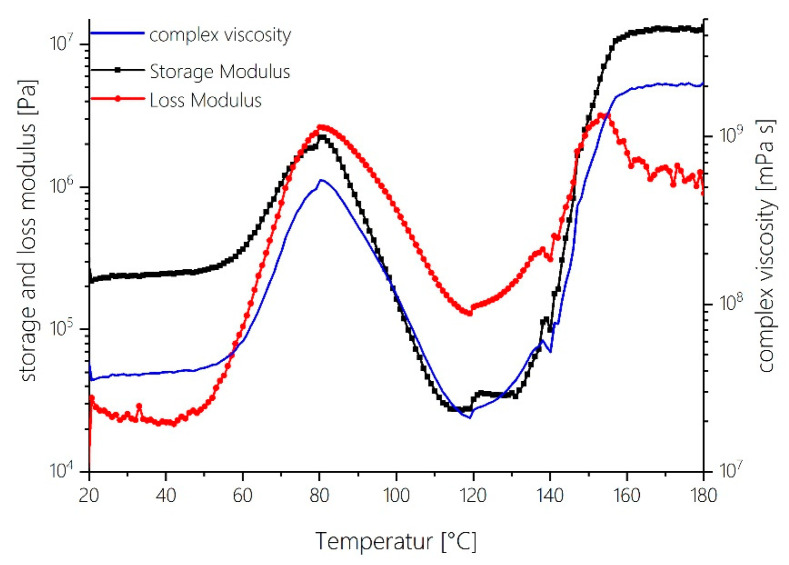
Changes in viscosity, storage modulus (G′), and loss modulus (G″) and complex viscosity ∣η*∣ during MF resin cure in dependence of temperature as recorded using a plate/plate rheometer setup at a heating rate of 2 °C min^−1^.

Rheological measurements of the spray-dried MF pre-polymer are shown in Figure 6. The reason for the increase in the storage modulus and the complex viscosity in the temperature range from 60–80 °C might be due to changes in the packing density of the powder. First there is a loose powder within which the individual grains can move relatively undisturbed, but as the temperature increases the grains start to pack together and the powder becomes stickier. This causes a higher resistance against the mechanic force. There is a point of intersection of the storage and the loss modulus at about 70 °C. After this intersection from about 80 °C on, the complex viscosity decreases, which indicates a softening of the powdered resin. The low viscosity is also in agreement with the relatively large proportion of the network component “OL”, which is characterized by a high proportion of free methylol groups, i.e., non-crosslinked functional groups. In this phase of dynamic thermal curing, the overall system thus consists of relatively flexible network components. The reaction partners are comparatively mobile and can orient themselves towards each other. Hence, functional groups can react not only with reaction partners in their immediate vicinity but also with ones that are located further away.

Curing during the moderate temperature regime from 60 to 110 °C hence proceeds via formation of a flexible network system which is mainly bridged by ether bonds.

**PHASE III**—Temperature range from around 110 to 130 °C

In the temperature range between 110 and 130 °C, essentially further conversion of methylol groups to ether bridges takes place. This can be clearly seen from the first derivative of the concentration profiles of the three spectroscopic components shown in Figure 3b. From a temperature of about 110 °C on, both the relative proportion of ether bridges-rich network components (spectroscopic concentration of the network component ET, Figure 3a) and the relative rate of ET formation (Figure 3b) increase significantly. At the same time, the spectroscopic concentration of the OL component decreases very strongly (Figure 3a). The maximum spectroscopic concentration of network segments rich in ether functionalities is reached at about 130 °C.

This change in the network structure at temperatures just below 110 °C towards a higher degree of crosslinking can also be seen from the dynamic DSC thermogram shown in Figure 5. At this temperature a significant increase in the heat capacity is visible. This exothermic effect can be attributed to the crosslinking reaction taking off at this point. It is the temperature on-set of the exothermal curing reaction as usually referred to in thermal analysis.

The progress in crosslinking results in a more rigid network structure as evident from the temperature-dependent viscosity profile shown in Figure 6. Up to approx. 110 °C, softening of the MF powder due to an increase in temperature takes place. From around 110 °C on, solidification due to cross-linking increasingly predominates and the complex viscosity after passing a minimum at approximately 120 °C gradually starts to increase. Considering the increased formation of ether bridges, the observed increase in viscosity is easily understood. In this phase, a more cross-linked and stiffer network still mainly based on ether bridges is formed.

**Figure 7:**
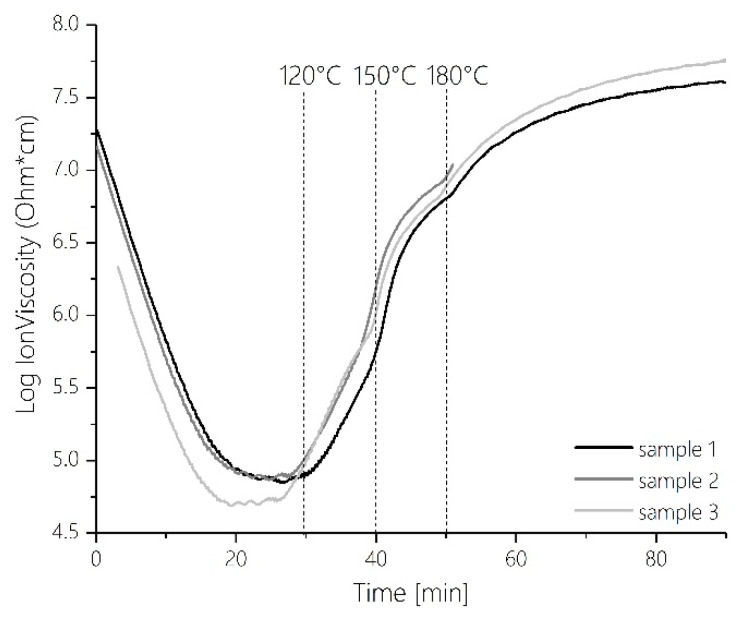
Time-dependent profile of ion viscosity during MF resin cure as measured by dielectric analysis.

The formation of a more strongly bridged and thus more rigid network structure is also visible in corresponding DEA measurements. Figure 7 shows the course of the ion viscosity over time in a dynamic measurement applying the same temperature ramp. The strong increase of the ion viscosity after about 25 to 30 min can be assigned to a temperature of about 110–120 °C on the temperature axis. This temperature range also includes the strongest relative rates of change in the relative proportions between the two spectroscopic components ET and OL (Figure 3b).

The essential chemical reactions that dominate the dynamics of network formation and that mainly take place in the temperature range between 110 and 130 °C can therefore be summarized schematically as the condensation of methylol groups to ether bridges (Equation (3)):

(3) R-NH-CH_2_-OH + HO-CH_2_-NH-R ⇄ R-NH-CH_2_-O-CH_2_-NH-R + H_2_O

The dominating spectral fingerprints in this phase of curing can be attributed (1) to structural elements that are rich in ether groups (to an increasing extent with increasing temperature) and (2) to methylol groups (to a decreasing extent with increasing temperature).

While in thermal and rheological as well as in dielectric measurements only phenomenological observations can be made in terms of increases in heat capacity, viscosity and ion viscosity respectively, the combined application of FTIR spectroscopy and Multivariate Curve Resolution (MCR) reveals not only the general increase in network density (in terms of increased formation of bridges and reduction in the number of free functional groups) but also the particular chemical nature of the functional groups that are mainly present in the forming thermoset network (ether bridges). Thereby structural information is available that is not accessible by, for instance thermal or rheological analysis.

The maximum proportion of ether-rich network components is found at a temperature of 130 °C. Therefore, we have defined 130 °C as the upper limit of the temperature range for Phase III of MF curing. During the elevated temperature regime within the interval from 110 to 130 °C, the formation of a more rigid, still mainly ether-bridged network structure takes place.

**PHASE IV**—Temperature range from around 130 to 170 °C

We have seen that in the temperature range from approx. 110 °C to >150 °C the ET-component shows the largest spectroscopic concentrations, i.e., the quantitatively most important fraction of network components bears ether bridges as a characteristic structural feature and the condensation of methylol to ether groups is the dominant reaction type.

From a temperature of about 120 °C onwards, the formation of methylene bridges becomes increasingly relevant. Figure 3a,b show that at about 120 °C both the rate of formation (Figure 3b) and the relative proportion (Figure 3a) of methylene bridge rich moieties of the MF network start to increase. This trend becomes particularly pronounced above 130 °C. At this temperature, the rate of methylene bridge formation already outweighs the rate of ether bridge formation (intersection point of the ET and MN concentration profiles in the first derivative of the relative spectroscopic concentration profiles in Figure 3b). The preferred formation of methylene bridges in this temperature range is also in agreement with the literature [30]. The dominant reactions in the phase of curing from 130 °C onwards can therefore, be summarized schematically with the following reaction equations

(4) R-NH-CH_2_-OH + H_2_N-R ⇄ R-NH-CH_2_-NH-R + H_2_O

(5) R-NH-CH_2_-O-CH_2_-NH-R ⇄ R-NH-CH_2_-NH-R + CH_2_O

(6) R-NH_2_ + CH_2_O ⇄ R-NH-CH_2_-OH

While the formation rate of the ether-dominated network components is at a maximum in the range between 110 and 120 °C, the formation rate of the methylene bridges is comparatively highest in the higher temperature range above 130 °C. The reaction of methylols with free amino groups is predominant and methylene bridges are formed (Equation (4)). Ether bridges are converted into methylene bridges (Equation (5)). The latter can only take place by splitting off formaldehyde (Equation (6)). The release of volatile components such as water and formaldehyde is observed in thermogravimetric analysis in this temperature range (Figure 4 TGA). Thus, their availability in the chemical equilibria (Equations (5) and (6)) is plausible. It must be assumed that the released formaldehyde either escapes from the system or reacts with melamine directly to form new methylols. These methylols in turn react mainly with neighboring amino groups directly to form methylene bridges according to Equation (4). This is also reflected in the course of the first derivative of the relative concentration profiles in Figure 3b for the OL component: from 130 °C onwards, the relative decrease of methylol functionalities occurs more slowly than at lower temperatures. This also makes sense from the point of view of the further solidification of the network: Due to the increasing immobility of the entire network, reactions in the local neighborhood of functional groups (so-called “next neighbor reactions”) occur more frequently from a temperature of 130 °C onward. This is also reflected in the rheological measurements, as the complex viscosity starts to rise sharply from 130 °C (Figure 6). The reaction partner of a methylol group will increasingly be an amine, which is more likely to be found in its immediate vicinity than another methylol unit is. This explains why no new ether bridges are formed in this temperature regime.

The chemical processes that dominate in the temperature interval between 130 and 150 °C are thus mainly the condensation of methylol units to methylene bridges and the conversion of ether bridges to methylene bridges. The rate of formation of methylene functionalities in the network reaches a maximum at 150 °C. At the same time, the decrease of the concentration of ether bridge-rich entities passes a maximum at 150 °C (as shown as a “negative maximum” in the first derivative in Figure 3b). Moreover, the contribution of the condensation of free methylol groups with melamine to the formation of methylene bridges is successively declining until the transformation of methylols into methylene bridges finally approaches zero.

It is remarkable that at this point of maximum reaction rate of methylene bridge formation, in the DSC, DEA and rheological profiles also notable features are present at the corresponding points of the reaction courses. In the DSC trace (Figure 5), a small maximum of the reaction enthalpy is visible at 150 °C. With the multivariate statistical analysis of the FTIR reaction spectra, this maximum can be assigned to the maximum formation rate during the exothermic curing via formation of methylene bridges. A reproducible abrupt increase in ion viscosity can also be detected at 150 °C meaning that the network at this point experiences a strong increase in rigidity (Figure 7). This corresponds well with rheological measurements. As indicated by the progression of viscosity as a function of temperature, the gel point is reached at a reaction temperature of 150 °C (compare the point of intersection of storage modulus and loss modulus in Figure 6).

The participation of free methylol groups in the reaction decreases steadily, and the transformation of methylols is no longer the main reaction leading to crosslinking and formation of bridges. From a temperature of >160 °C on, the conversion of free methylols practically comes to a halt.

At very high temperatures >170 °C the mobility of the network steadily decreases further as the cross-linking progresses (Figure 6 and Figure 7). In the course of the DEA trace, a further sharp increase in ion viscosity can be seen at 170 °C. Since this increase also marks the end of the reaction of methylol groups, the temperature of 170 °C was chosen to mark the end of PHASE IV in the curing of MF. At this point, the network has become too rigid for reactions of the methylols to occur.

It should be noted that at this point the exothermal curing signal in the DSC trace comes to an end (T_end_ = 170 °C). From 170 °C on, practically no exothermic enthalpy can be detected at this heating rate (Figure 5). By definition, the degree of conversion would be 100% at this point for kinetic evaluations based on thermal analysis. IR spectroscopic analysis, however, shows that the chemical conversion processes are still far from complete. Further reaction still occurs during Phase V even after reaching the gel point. Therefore, it would be wrong to trust DSC at this point and assume „complete turnover“. Rather, it means that the chemical transformations occurring during Phase V of the curing process cannot be observed by DSC due to lack of sensitivity.

**PHASE V**—Temperature range >170 °C

In the temperature range above 170 °C (starting from about 160 °C on), the relative proportion of network components carrying a high number of methylol groups approaches a minimum value that does not change significantly with further temperature increases (Figure 3a). The rate of change of the OL component rapidly approaches zero from this temperature onwards. This means that none of the remaining free methylol functions present at this point of curing are still involved in further condensation reactions. Neither new ether bridges nor new methylene bridges are formed from methylols. The reason for this is that the methylols are “entrapped” within the rigid network structure and are no longer able to react because the network has solidified and no reaction partner can be found in their immediate vicinity. The complex viscosity has reached its maximum value and stays nearly constant in this high temperature range (Figure 6). This makes sense, as no net change of the overall amount of cross-links takes place any more.

It is important to note, however, that the amount of free methylol functionalities present in the network is not zero. According to the spectroscopic fingerprints, free methylols are still present in the cured resin in its final state. The presence of methylol groups in “fully” cured MF samples has also been shown in a previous study by solid-state ^13^C-NMR spectroscopy where the characteristic chemical shifts of methylol-carbons were found [19]. This means that a so-called “fully cured resin” cannot, strictly speaking, be said to have a condensation degree of 100% as long as by this is meant that all reactive groups capable of condensation should have reacted at this point.

In contrast, at temperatures >170 °C, a further decrease in the ether-bridged components of the network takes place. From 170 °C on, only the net conversion of ether bridges into methylene bridges occurs and a consolidation of the rigid network takes place. At this stage, only the conversion of ether bridges into methylene groups is still possible within the rigid thermoset network, since one and the same bridge only rearranges itself by splitting off formaldehyde. The network consolidates to a certain extent under formation of methylene bridges which are known to be more stable at higher temperatures [30]. With further increasing temperature, the rearrangement of the ether bridges into methylene bridges still occurs but with declining intensity. The chemical processes taking place during Phase V can thus be summarized in a simplified way, again, by reaction Equations (5) and (6). The liberated formaldehyde can either react again immediately via the reaction sequence Equation (6) → Equation (5) or escape from the system.

In the high temperature regime above 170 °C, consolidation of the thermosetting network takes place. At this temperature, the system is well beyond gel point and mobility is very restricted. The only reaction within this rigid solid is the transformation of ether-bridges to methylene bridges.

### 3.4. Visualisation of the Reaction Scheme of MF Cure in the Solid State

Based on the chemometric and kinetic evaluation of the infrared spectra obtained in real-time during thermal cure we identified five subsequent phases of MF curing in the solid state.

PHASE I. At this stage, the pre-polymer system is cross-linked only to a very minor degree. The most abundant functional groups are free methylols. During this phase there are no spectroscopically detectable changes in the network composition and no net chemical transformations of individual network components. This first phase of curing could be briefly described as stationary phase (Phase I “stationary phase”).

PHASE II. In this second phase of slightly increased curing temperature, a network is formed and the first significant increase in degree of cross-linking occurs. Since this network consists mainly of ether bridges formed from methylols and methylene bridges and the resulting network still has a reasonably high degree of mobility, this temperature range of dynamic curing of MF can also be called the phase of flexible network formation (Phase II, “flexible network formation”).

PHASE III. In this phase of MF curing, the network is further strengthened. Almost exclusively free methylol groups are converted into ether bridges by condensation. The mobility of the entire system decreases sharply and there is a further increase in the degree of cross-linking and viscosity. During this phase, the mobility of the functional groups continues to become increasingly restricted. The relative proportion of ether bridges is greatest in this phase. Therefore, we propose to speak of a rigidification of the network (Phase III: “rigid network formation”, or “network rigidification”).

PHASE IV. In this phase of MF curing, part of the remaining methylol groups reacts to form methylene bridges, as far as this is allowed by the solidified network, which is by now strongly restricted in its mobility. The ether bridge-dominated network starts to change into a system of methylene bridges. The more flexible bridge (RN-CH_2_-O-CH_2_-NR) on a molecular scale changes into a shorter, more rigid bridge (RN-CH_2_-NR). This leads to a further hardening of the network. The gel point is also reached within this curing phase. Thus, we propose to speak of Phase IV as network hardening (Phase IV, “network hardening”). During the elevated temperature regime between 130 and 170 °C, a rigid mainly methylene-bridged network is formed from transformation of both ether bridges and consumption of methylols. The temperature of 170 °C marks the end of Phase IV of the curing process.

PHASE V. In the final phase of curing, which takes place at high temperatures above 170 °C, only the conversion of ether bridges into methylene bridges takes place. There is no further conversion of the methylol groups that are still present at this stage. Their proportion and distribution in the formed thermoset network is to a certain extent “frozen” and fixed at this temperature. In the context of Phase V of MF curing, we speak of network consolidation (Phase V “network consolidation”), because a large part of the more hydrolysis-sensitive groups (ether bridges) are converted into more stable methylene bridges. The bridging state finally achieved at this stage defines the corresponding technological property profile of the resulting cured resin.

**Figure 8:**
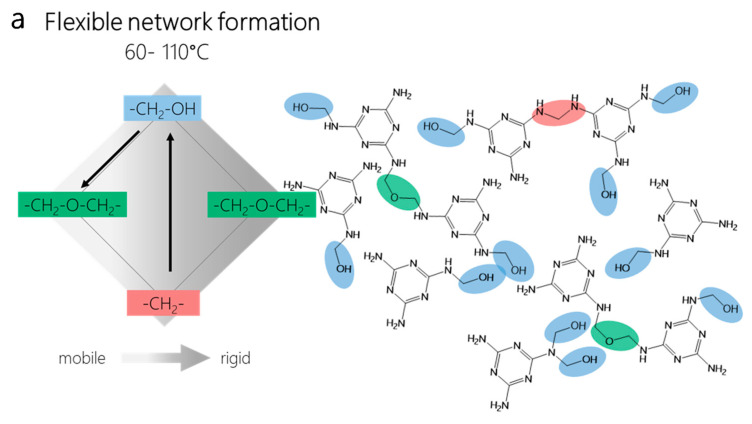

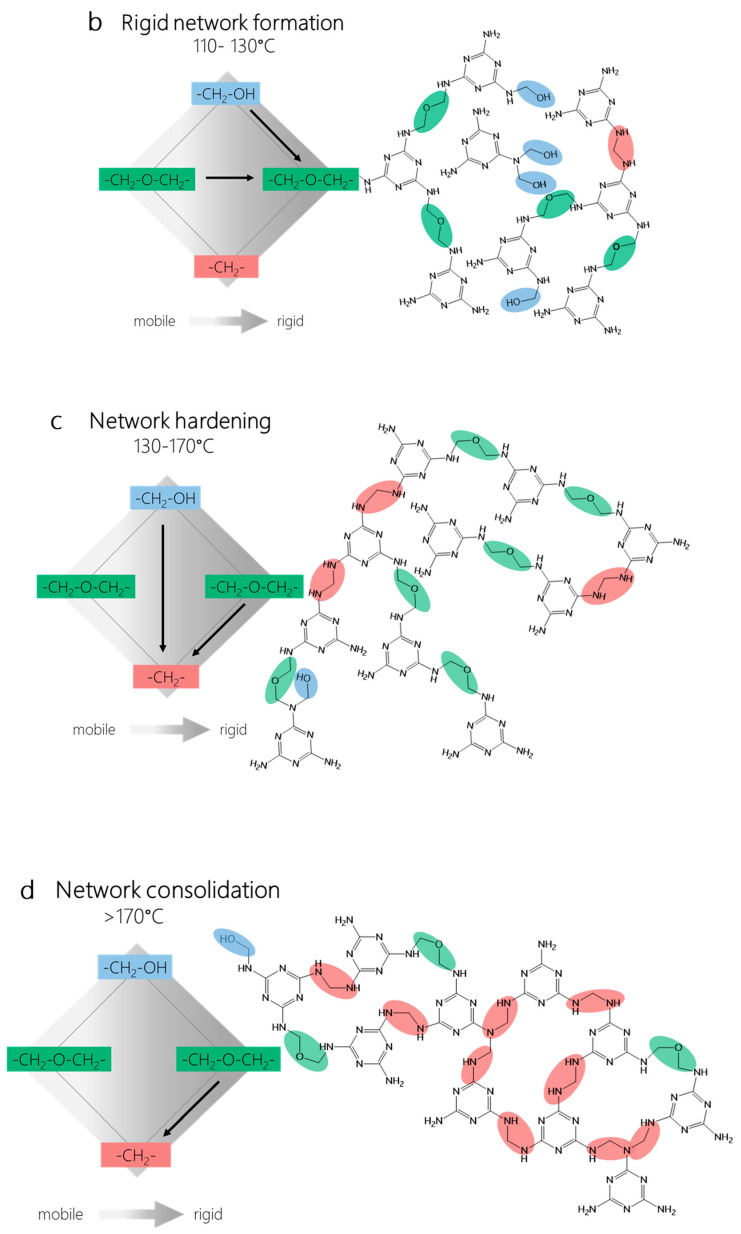
The phases of solid MF resin cure during which the major chemical transformations take place. (**a**) Flexible network formation, taking place in a temperature range between 60 and 110 °C; (**b**) Rigid network formation, taking place in a temperature range between 110 and 130 °C, (**c**) Network hardening, taking place in a temperature range between 130 and 170 °C; and (**d**) Network consolidation, taking place in a temperature range higher than 170 °C. The color gradient from light to dark grey in the diamond from left to right indicates the increasingly solidifying network. The black arrow in (**b**) between the ether bridges indicates no chemical transformation but the transition of a rather mobile, ether-dominated into a rather rigid ether-dominated network structure via network stratification. In other words, the transition from a multitude of interpenetrating network fragments into a more homogenous, cross-linked larger network of greater rigidity.

Figure 8 summarizes the reaction phases (Phase 2–5) where net chemical transformations take place.

## 4. Conclusions

In the present study, the main focus of the work was to find a mechanistic framework that allows an in-depth modelling of the hardening process in the solid state on the basis of non-destructive and real-time ATR FTIR/MCR analysis. Therefore, the objective of the present study was not so much to simulate an industrial curing process as realistically as possible in the laboratory, but rather, in a first step, to provide under more or less idealized conditions, a method that (1) characterizes the typical chemical transformations occurring during MF resin cure in the solid state and (2) allows modelling and, in the end, tailoring resin cure to yield defined cross-linked resin states. In order to evaluate the performance of the method, we have therefore made the following main simplifications in comparison to industry: (1) We have investigated a powder resin to experimentally exclude the disturbing influences of water signals (i.e., eliminate superposition of vibration signals and dilution effects caused by evaporating solvent due to the applied heating gradient during the measurement) and to be able to follow the chemical processes during hardening of the resin in the infrared spectral region with satisfactory accuracy. (2) Compared to industrial processes, a rather low heating rate of 2 °C min^−1^ was used as the temperature gradient for the dynamic curing experiments in the present study for different reasons: (a) to follow the curing process on a reasonable time scale, (b) to obtain a good experimental fit between the spectroscopic data and the thermal, rheological and dielectric reference methods, and (c) to get a good signal-to-noise ratio for the spectral analysis within the time required for data acquisition. (3) Furthermore, in contrast to industrial processes, the curing tests were carried out without presence of any catalyst to maintain the curing rate within a well-analyzable time regime and to exclude catalyst specific effects.

Based on chemometric analysis of the infrared spectroscopic data we have found, that the hardening of MF resin can, in principle, be subdivided into five successive phases: After a low temperature phase (“stationary phase”, Phase I) where no net changes in chemical composition occurs, the formation of a flexible, ether-bridges-based network takes place (“flexible network formation”, Phase II). Then, the network increases in cross-link density while the number of ether bridges further increases (“rigid network formation”, Phase III). Thereafter, methylol functions and ether bridges transform into a mainly methylene-bridge based network. The system passes the gel point and methylol transformation comes to a halt (“network hardening”, Phase IV). Finally, only ether bridges are further transformed into methylene bridges at temperatures beyond 170 °C (“network consolidation”, Phase V). The basis for this classification was the course of the relative proportions of “spectral components”, which were extracted by MCR from the entirety of the reaction spectra recorded in situ during hardening. Their spectral fingerprints were assigned to functional group vibrations. The spectroscopically determined curing phases agree well with complementary information obtained from thermal, rheological and dielectric measurements and provide a consistent picture of the processes taking place during curing in the solid state. Due to the good agreement of the different methods, it is assumed that the FTIR/MCR based model adequately describes the curing process and a satisfactory characterization of the forming and hardening thermoset network was achieved on a molecular level.

The present work has various implications. Based on our observations, we consider it important to point out that the common descriptions of the final state of a thermoset polymer network are problematic in the case of MF. The final state of a thermoset cured under certain conditions is usually given with reference to a conversion degree of 100%. This state of complete cure can be determined by thermal, spectroscopic or other methods. The DSC-based definition uses the enthalpy integral of the exothermic curing peak in the DSC trace as a reference point. From our point of view, this can be problematic for MF systems, because it looks like the final phase of network consolidation is not detected with sufficient sensitivity by DSC. Since in Phase V, chemical transformation of ether to methylene bridges takes place and these groups differ significantly in their, for instance, hydrolytic stability against steam, this phase of network consolidation is considered to be crucial for the emergence of the final surface film properties.

An endpoint of 100% conversion based on spectroscopic measurements is often related to the complete consumption of a specific functional group. In polyurethanes, for example, the disappearance of the isocyanate band may be monitored in the IR. With isocyanates, this can even be done univariately since there are no interfering spectroscopic signals. However, such a definition is problematic for MF resins for two reasons. Firstly, no such isolated absorbance band can be found whose conversion could be easily tracked and secondly, in principle, there is no functional group that is completely transformed.

An alternative heuristic definition of complete conversion is to use as a reference point the point at which no further changes are observed in the spectroscopic signals of the curing resin. However, this is unsatisfactory in that resins with different properties can be produced under different curing conditions. These differences in properties are due to different chemical states in the resin that may be spectroscopically stable in the above sense. Therefore, reference to the point of invariability of the spectral signal alone does not necessarily help in discussing the properties of the cured resin.

We therefore suggest that the latter heuristic criterion should be supplemented as follows: an MF resin should be considered fully cured once a stable ratio between the three spectral components OL, ET and MN has established and no further transformation of the three components into each other can be detected. This specific ratio can then be used to summarize the chemical state of this resin at 100% conversion. Thus, the proportions between the spectroscopic components can, in principle, be used with advantage to provide the basis for meaningfully modelling and predicting the technological properties of cured MF resins based on molecular information.

A future approach to tailor the property profiles of cured melamine resin surfaces based on molecular descriptors as presented here would proceed by (a) first systematically determining the influence of curing conditions (and ultimately, the influence of synthesis conditions and further processing steps such as storage, drying, impregnation, etc.) on the relative OL:ET:MN ratio (influence of process factors) and (b) determining the relationship between the OL:ET:MN ratio and technologically relevant quality characteristics of the cured MF resin. This would enable a target-oriented technological functionality design of melamine resin surfaces based on the molecular understanding of the underlying chemical system.
